# The US National Tuberculosis Surveillance System: A Descriptive Assessment of the Completeness and Consistency of Data Reported from 2008 to 2012

**DOI:** 10.2196/publichealth.4991

**Published:** 2015-10-15

**Authors:** Rachel S Yelk Woodruff, Robert H Pratt, Lori R Armstrong

**Affiliations:** ^1^ Centers for Disease Control and Prevention National Center for HIV/AIDS, Viral Hepatitis, STD, and TB Prevention Division of Tuberculosis Elimination Atlanta, GA United States

**Keywords:** public health surveillance, disease notification, information systems, data cleaning, quality assurance

## Abstract

**Background:**

In 2009, the Tuberculosis (TB) Information Management System transitioned into the National TB Surveillance System to allow use of 4 different types of electronic reporting schemes: state-built, commercial, and 2 schemes developed by the Centers for Disease Control and Prevention. Simultaneously, the reporting form was revised to include additional data fields.

**Objective:**

Describe data completeness for the years 2008-2012 and determine the impact of surveillance changes.

**Methods:**

Data were categorized into subgroups and assessed for completeness (eg, the percentage of patients dead at diagnosis who had a date of death reported) and consistency (eg, the percentage of patients alive at diagnosis who erroneously had a date of death reported). Reporting jurisdictions were grouped to examine differences by reporting scheme.

**Results:**

Each year less than 1% of reported cases had missing information for country of origin, race, or ethnicity. Patients reported as dead at diagnosis had death date (a new data field) missing for 3.6% in 2009 and 4.4% in 2012. From 2010 to 2012, 313 cases (1%) reported as alive at diagnosis had a death date and all of these were reported through state-built or commercial systems. The completeness of reporting for guardian country of birth for pediatric patients (a new data field) ranged from 84% in 2009 to 88.2% in 2011.

**Conclusions:**

Despite major changes, completeness has remained high for most data elements in TB surveillance. However, some data fields introduced in 2009 remain incomplete; continued training is needed to improve national TB surveillance data.

## Introduction

Tuberculosis (TB) incidence (or case notification) is used globally for monitoring trends, planning, and evaluating public health programs [1,2]. In the United States, national incidence reporting began in 1953, with documented cases and operational data from each reporting jurisdiction submitted in aggregate [3]. By 1985, all jurisdictions were reporting individual cases using a standardized form, the Report of Verified Case of Tuberculosis (RVCT) [4]. In 1993, the RVCT was expanded to include additional risk factors and laboratory information, and TB surveillance data began to be entered and transmitted to the Centers for Disease Control and Prevention (CDC) through a single software system [5].

The US National Tuberculosis Surveillance System (NTSS) underwent major revisions in 2009 [6]. RVCT was expanded to include 11 new data fields, and 25 of 38 existing fields were modified. Concurrently, state and local reporting areas transitioned from reporting TB case data through the Tuberculosis Information Management System (TIMS), a stand-alone, modem-based system developed at the CDC, to their choice of 4 reporting schemes: (1) the National Electronic Disease Surveillance System (NEDSS)-base system, a CDC-developed infrastructure; (2) the electronic RVCT (eRVCT), also developed by the CDC; (3) state-developed custom software systems; or (4) commercial software developed by private companies. All reporting schemes were required to conform to specific Public Health Information Network and NEDSS data standards [7,8].

The transition from a single reporting scheme to a choice of different types of schemes allowed state and local TB programs more control over the structure of their surveillance systems and gave them responsibility for their own data validation [9]. Prior to 2009, surveillance data came to the CDC via TIMS, which had a built-in data validation system for alerting logic errors to help ensure accurate data entry and reporting. These validation standards were retired with TIMS in 2010, although the CDC-developed eRVCT and NEDSS-base system retained validation rules similar to those in TIMS. Validation rules for state-developed and commercial schemes vary by jurisdiction. Furthermore, routine maintenance, updates, changes, and enhancements of state-developed and commercial reporting schemes are now at the expense of state and local TB programs; information technology (IT) expertise is necessary at the state and local level to maintain and update these types of systems [9]. Modifications of state and commercial reporting schemes, such as changes in RVCT data fields, have to be done at the level of the individual reporting jurisdiction; therefore, modifications to NTSS are more complicated than they were prior to 2009, when the CDC was able to update a single system and provide all reporting jurisdictions with updated software that incorporated the revisions.

The objectives of this report are to describe the completeness and consistency of TB case data reported to the CDC from 2008 to 2012, to determine the extent to which the 2009 changes in RVCT and reporting schemes affected the data, and to find ways to improve data quality. Although the surveillance report and the reporting schemes described here are specific to TB, the analytical methods and results may be useful to managers of other public health programs who are contemplating similar changes in surveillance systems or reporting schemes.

## Methods

### Data sources

NTSS receives TB surveillance data electronically from the 50 states and the District of Columbia [6]. The reporting officials in TB programs collect laboratory and clinical TB data from a variety of sources and store them in electronic reporting systems. From 1998 to 2009, those officials submitted TB surveillance data through TIMS by using file-transfer protocol and controlled-access Internet and modem transfer [10]. Starting in 2009, TB surveillance data have been transmitted using Public Health Information Network Messaging Service software in HL7 messaging format.

The CDC provides preliminary TB surveillance datasets weekly for reporting program officials to verify reported data. The CDC creates final TB surveillance datasets annually for reporting, research, and publications. Since 2009, TB data reported to the CDC have been subjected to a data-cleaning routine before a finalized dataset is created. The data cleaning routine is applied to selected data fields using a hierarchical strategy as determined by CDC staff (eg, a dependent field, such as the year of previous TB episode, is deleted if the independent field, such as history of previous TB, is not present) that creates a dataset that has fewer inconsistencies but not necessarily more accuracy. Our analysis included only clean, finalized annual datasets.

### Analysis

We examined responses from NTSS data elements from 2008 to 2012 (the most recent year of data at the time of analysis) and new elements from 2009 to 2012. Although NTSS includes data from 1993 to 2012, the purpose of this study was to examine how the changes in data elements and reporting schemes affected the data; therefore, the study period begins the year before the changes occurred. New data elements from Alaska, California, Connecticut, Illinois, Missouri, Mississippi, North Carolina, North Dakota, New York City, and Ohio were not included for 2009 because these jurisdictions used TIMS that year and the new elements were not supported. In addition, we excluded California and Vermont from analyses that included HIV test results for 2008-2012 because HIV reporting practices were different for these jurisdictions.

Reporting jurisdictions were categorized according to the type of reporting scheme (TIMS, commercial, eRVCT, NEDSS-base, or state-developed) used in 2009 and 2010-2012. Because of the changes in both reporting schemes and RVCT in 2009, data from that year were examined separately from latter years’ data.

Data were categorized into subgroups and data elements associated with subgroups were assessed for completeness (eg, the percentage of patients dead at diagnosis who had a date of death reported) and consistency (eg, the percentage of patients alive at diagnosis who erroneously had a date of death reported). The results are presented for a subset of data elements that are clinically or demographically important or exhibited inconsistency or incompleteness in reporting. Furthermore, for each TB case we selected key data elements from 3 different categories: risk factors, clinical aspects of TB disease, and molecular aspects of TB disease.

## Results

From 2008 to 2012, 56,040 cases were reported to NTSS [6]. Each year, fewer than 1% of reported cases had missing or unknown information for origin of birth (nativity; 59/56,040), or race/ethnicity (197/56,040). One data element that demonstrated inconsistency in completeness was correctional facility status (residence in correctional facility at time of diagnosis), for which 6.5% of cases (746/11,520) had unknown or missing information in 2009, compared with approximately 1% or less of cases (265/44,529) in other years (Table 1). When correctional facility status was examined by reporting system (Table 2), information was missing for 17.1% (729/4266) of the cases reported by jurisdictions using TIMS in 2009, while the other reporting systems had less than 1% of cases (17/6871) missing for this element. Among cases reported as residents in correctional facilities at the time of diagnosis, information on the type of correctional facility was missing for 9% (10/110) of cases reported through state-developed reporting systems in 2009 and 2010-2012 (25/267), compared to less than 3% (17/1386) through TIMS, commercial, NEDSS-based, and eRVCT reporting systems for those same years (Tables 2 and 3).

**Table 1:** Completeness of trend data elements reported to the National Tuberculosis Surveillance System, United States, 2008-2012.

		2008		2009		2010		2011		2012	
		N	%	N	%	N	%	N	%	N	%
Total reported TB cases		12,904	100.0	11,520	100.0	11,163	100.0	10,517	100.0	9945	100.0
**Resident in correctional facility**											
	Yes	499	3.9	465	4.0	489	4.4	423	4.0	386	3.9
	No	12,386	96.0	10,309	89.5	10,536	94.4	10,036	95.4	9509	95.6
	Unknown/ Missing	19	0.1	746	6.5	138	1.2	58	0.6	50	0.5
Type of correctional facility indicated^a^		492	98.6	451	97.0	471	96.3	411	97.2	382	99.0
History of TB		572	4.4	492	4.3	510	4.6	511	4.9	481	4.8
Year of TB reported^b^		564	98.6	455	92.5	493	96.7	497	97.3	462	96.0
Year of TB missing^b^		8	1.4	37	7.5	17	3.3	14	2.7	19	4.0
Initial DST done^c^		9604	98.4	8725	98.2	8316	98.4	7966	98.5	7315	96.3
Isoniazid results^c^		9385	97.7	8684	99.5	8279	99.6	7923	99.5	7258	99.2
Rifampin results^c^		9377	97.6	8678	99.5	8279	99.6	7919	99.4	7260	99.3

^a^Among patients who were residents of correctional facilities at the time of diagnosis.

^b^Among cases that reported history of previous TB.

^c^Drug susceptibility test. Among patients who had positive culture; includes resistant and susceptible test results.

**Table 2:** Completeness and consistency of data elements reported to the National Tuberculosis Surveillance System by type of reporting system, United States, 2009.

		TIMS^b^		Commercial		State developed		NEDSS-base^c^		eRVCT^d^	
		N	%	N	%	N	%	N	%	N	%
Total reported TB cases		4266	100.0	470	100.0	2815	100.0	2852	100.0	1117	100.0
**Resident in correctional facility**											
	No	3438	80.6	463	98.5	2689	95.5	2654	93.1	1065	95.3
	Unknown/ Missing	729	17.1	0	0.0	16	0.6	1	0.0	0	0.0
	Yes	99	2.3	7	1.5	110	3.9	197	6.9	52	4.7
Type of correctional facility indicated^a^		92	92.9	7	100.0	100	90.9	197	100.0	51	98.1
History of TB		222	100.0	20	100.0	130	100.0	89	100.0	31	100.0
Year of TB reported		195	87.8	18	90.0	124	95.4	88	98.9	30	96.8
Year of TB missing		27	12.2	2	10.0	6	4.6	1	1.1	1	3.2
Alive at time of TB diagnosis		4182	100.0	466	100.0	2747	100.0	2787	100.0	1094	100.0
Date of death indicated		N/A	N/A	8	1.7	40	1.5	0	0.0	0	0.0
Patient < 15 years of age		N/A	N/A	32	100.0	153	100.0	214	100.0	44	100.0
Guardian country of birth		N/A	N/A	28	87.5	119	77.8	189	88.3	36	81.8
Patient 15 years of age or older		N/A	N/A	438	100.0	2662	100.0	2638	100.0	1073	100.0
Guardian country of birth		N/A	N/A	1	0.2	63	2.4	8	0.3	2	0.2

^a^Among patients who were residents at correctional facilities at the time of diagnosis.

^b^Tuberculosis Information Management System.

^c^National Electronic Disease Surveillance System.

^d^Electronic Report of Verified Case of Tuberculosis.

**Table 3:** Completeness and consistency of data elements reported to the National Tuberculosis Surveillance System by type of reporting system, United States, 2010-2012.

	Commercial		State developed		NEDSS-base^a^		eRVCT^b^	
	N	%	N	%	N	%	N	%
Total reported TB cases	12,685	100.0	8551	100.0	7646	100.0	2743	100.0
Resident in correctional institute	312	100.0	267	100.0	489	100.0	230	100.0
Type of correctional facility indicated	303	97.1	242	90.6	489	100.0	230	100.0
History of TB	689	100.0	421	100.0	286	100.0	106	100.0
Year of TB	665	96.5	408	96.9	276	96.5	103	97.2
Alive at time of TB diagnosis	12,397	100.0	8330	100.0	7468	100.0	2680	100.0
Date of death indicated	162	1.3	151	1.8	0	0.0	0	0.0
Patient < 15 years of age	597	100.0	484	100.0	504	100.0	119	100.0
Guardian country of birth	525	87.9	374	77.3	485	96.2	105	88.2
Patient 15 years of age or older	12,088	100.0	8067	100.0	7142	100.0	2624	100.0
Guardian country of birth	38	0.3	317	3.9	19	0.3	3	0.1

^a^National Electronic Disease Surveillance System.

^b^Electronic Report of Verified Case of Tuberculosis.

In 2009, 7.5% of cases (37/492) with a previous history of TB reported were missing the year previous TB disease occurred, compared to 1.4% (8/572) in 2008 (Table 1). No previous year of TB disease was reported for cases that did not have a history of previous TB disease indicated. Among cases reported in 2009 with a previous history of TB disease indicated, the highest percentage of missing years of previous TB disease was with TIMS at 12.2% (27/222; Table 2), compared to 10% or less (10/270) of cases with a previous history of TB that were missing years of previous TB disease reported through the other systems (Table 2). For 2010-2012, the year of previous TB disease was missing for 3-4% of cases (50/1502) for which previous TB disease history was indicated across all reporting system types (Table 3).

Of the 426 culture-positive cases reported in 2008 that did not have initial drug susceptibility testing (4.2% of all culture-positive cases, 426/10,024, including those with unknown or missing initial drug susceptibility test results), 1 case was reported as susceptible to isoniazid and 1 case was reported as susceptible to rifampin. From 2009 to 2012, no culture-positive cases without initial drug susceptibility test reported “done” had isoniazid or rifampin results reported. For sputum culture and sputum smear results reported as negative or positive, over 99% of cases (31,098/31,410) each year had a sputum smear or sputum culture collection date reported (Table 4). No sputum culture or sputum smear collection dates were reported for cases that did not have an associated sputum culture or sputum smear test done.

**Table 4:** Completeness of new data elements reported to the National Tuberculosis Surveillance System, United States, 2009-2012.

	2009		2010		2011		2012	
	N	%	N	%	N	%	N	%
Dead at time of TB diagnosis	160	100.0	252	100.0	245	100.0	221	100.0
Date of death indicated	153	95.6	244	96.8	235	95.9	213	96.4
Sputum culture positive or negative	5743	100.0	9018	100.0	8599	100.0	8050	100.0
Sputum collection date indicated	5704	99.3	8952	99.3	8502	98.9	7940	98.6
Sputum smear positive or negative	5788	100.0	9162	100.0	8709	100.0	8217	100.0
Sputum smear date indicated	5743	99.2	9139	99.7	8674	99.6	8141	99.1
Patient < 15 years of age	443	100.0	637	100.0	578	100.0	489	100.0
Guardian country of birth	372	84.0	555	87.1	510	88.2	424	86.7
Lived outside US > 2 months	120	27.1	161	25.3	124	21.5	139	28.4
Country where lived indicated^a^	116	96.7	151	93.8	119	96.0	135	97.1

^a^ Among pediatric patients who lived outside the country for 2 months.

For cases reported as dead at TB diagnosis, 4.4% (7/160) were missing date of death in 2009, the first year date of death information was collected, and 4.6% (8/221) were missing it in 2012 (Table 4). In 2009, 48 of 7094 TB cases (0.70%) were reported as alive at diagnosis and had a date of death indicated (Table 5). A majority of these (83%, 40/48; Table 2) were reported through state-developed systems. From 2010 to 2012, 313 of 30,875 TB cases (1%) were reported as alive at diagnosis and had a date of death indicated (Table 5); all were reported through state-developed or commercial reporting systems (Table 3).

**Table 5:** Consistency between new data elements reported to the National Tuberculosis Surveillance System, United States, 2009–2012.

	2009		2010		2011		2012	
	N	%	N	%	N	%	N	%
Alive at time of TB diagnosis	7094	100.0	10,903	100.0	10,261	100.0	9711	100.0
Date of death indicated	48	0.7	111	1.0	116	1.1	86	0.9
Sputum culture not done/unknown/missing	1511	100.0	2100	100.0	1851	100.0	1748	100.0
Sputum collection date indicated	5	0.3	0	0.0	0	0.0	0	0.0
Sputum smear not done/unknown/missing	1466	100.0	1998	100.0	1799	100.0	1703	100.0
Sputum smear date indicated	1	0.1	0	0.0	0	0.0	0	0.0
Patient 15 years of age or older	6811	100.0	10,526	100.0	9939	100.0	9456	100.0
Guardian country of birth	74	1.1	102	1.0	152	1.5	123	1.3
Lived outside US > 2 months	101	1.5	178	1.7	183	1.8	157	1.7
Country where lived indicated^a^	92	91.1	168	94.4	179	97.8	155	98.7

^a^Among pediatric patients who lived outside the country for more than 2 months.

Country of birth of primary guardian, whether the patient lived outside the United States for more than 2 months and if so in what countries, are new data elements requested for pediatric patients (<15 years of age). Completeness ranged from 84% (372/443) in 2009 to 88.2% (510/578) in 2011 for the guardian country of birth for pediatric TB cases and from 93.8% (151/161) in 2010 to 97.1% (135/139) in 2012 for the country where the pediatric patient lived for more than 2 months (Table 4). Among nonpediatric cases (15 years of age and older), 1-2% (451/36,732) each year indicated a country of birth for the primary guardian. In 2009 and 2010-2012, completeness in reporting for guardian country of birth for pediatric TB patients was highest for those reported through NEDSS-base software systems (88.3%, 189/214, and 96.2%, 485/504, respectively; Tables 2 and 3). Nonpediatric cases with primary guardian information were predominantly reported through state-developed software systems in 2009 (Table 2) and 2010-2012 (Table 3).

## Discussion

### Principal Findings

Considering the extent of changes the US TB Surveillance System underwent in 2009, TB surveillance data have maintained a high level of completeness, with most data elements showing the same levels of completeness after 2009. New data elements, for which collection and reporting began in 2009 for most reporting jurisdictions, have varied completeness but show an overall improvement from 2009 to 2012. Some new data elements are taking longer to reach a high percentage of completeness at the state and local levels, or are less complete or less concordant in 2012 than they were in 2009. For example, patients who were dead at the time of TB diagnosis should have had a corresponding date of death recorded (the date-of-death data element was introduced in 2009). However, some jurisdictions reported a date of death for patients who were alive at diagnosis, which occurred more frequently in 2012 than in 2009 (Table 5). If a patient is alive at TB diagnosis and dies during therapy, there is no corresponding date of death field; therefore, some reporting jurisdictions may be recording the date of patient death in the field for death date of patients who were dead at the time of TB diagnosis. Among cases reported in 2009 that were alive at diagnosis and had a date of death recorded, 58% (28/48) had a date of death that matched the date therapy was stopped (data not shown), indicating that the date of death field was used to record the date of death during therapy. Completeness may also have been affected by lack of information or inability to find information in patient records, misinterpretation of data element definitions, or use of a paper reporting form that does not match the electronic reporting data entry form [2]. For some jurisdictions, electronic reporting systems may not have been revised to accommodate reporting of certain data elements; therefore, those elements cannot be reported electronically. Ongoing training of local staff to account for turnover and changes in duties may improve completeness of reporting [2].

The data cleaning routine does not take into consideration all possible data errors. Information requested specifically for all TB patients less than 15 years of age was sometimes reported for cases 15 years of age or older (Tables 2, 3, and 5), and the date of death may have been indicated for patients who were alive at diagnosis (Tables 2, 3, and 5); these discrepancies are not corrected as part of data cleaning. Therefore, care is warranted when working with NTSS data for reporting or research purposes. Proper subsetting is needed to prevent inclusion of patients who should not be included in a specific subset for analysis, such as patients alive at diagnosis when analyzing date of death, as these exclusions are not built into the dataset and omitting them could result in erroneous results.

Differences in completeness of data reported through the different electronic systems may be due to system configuration or reporting practices within the jurisdictions. The high percentage of missing correctional facility information reported in 2009 (Table 1) was due to data transmission problems experienced by a single reporting jurisdiction. The information for residence in a correctional facility existed in TIMS but was not transferred from TIMS to the jurisdiction’s new reporting system. Furthermore, commercial and state-developed reporting systems are responsible for their own validation, which could account for some higher percentages of missing or inaccurate data. TB case surveillance data do not allow for assessment of systems or reporting practices at the state and local level, so it was not possible to distinguish between factors related to systems or reporting practices in this analysis.

In 2009 there was an unexpected and significant decline in the numbers of TB cases reported to NTSS compared to previous years [11]. Changes to electronic reporting systems were not deemed to be a causal factor. Rather, we concluded that the decline in TB cases was a result of decreased TB diagnoses in the United States. Therefore, we did not consider the unexpected decline in TB cases in 2009 to be a factor in our study.

### Limitations

This study has several limitations. Limited resources prevented us from conducting a validation study at the local level to compare patient data from medical charts to the data reported to NTSS. This would have been especially valuable to assess data elements that exhibited inconsistency. The data-cleaning routine replaced some validation rules that existed in TIMS but may not have improved the quality of data reported to the CDC. For example, from 2009 to 2012, 2 cases reported as not having initial susceptibility testing done were also reported as susceptible to both isoniazid and rifampin (data not shown), indicating that initial drug susceptibility testing may actually have been done. Because the cases were reported as not undergoing susceptibility testing, the susceptibility results were deleted for these cases during data cleaning and therefore are not reflected in the clean, finalized dataset. Isoniazid and rifampin are important drugs for treating TB and resistance to both defines multidrug-resistant TB. If susceptibility testing was indeed done for isoniazid and rifampin, then drug susceptibility testing should be reported as “done” on RVCT.

### Conclusion

Several ongoing efforts have been implemented to improve the quality of surveillance reporting. The CDC initiated a series of trainings in 2010 with the goal of familiarizing state and local reporting jurisdictions with the updated RVCT and reporting requirements [12]. Additionally, in 2011, the CDC conducted a series of trainings on quality assurance of TB data [13]. The trainings culminated in a published manual that is available to reporting jurisdictions and others interested in attaining high-quality surveillance data [14]. A collection of reports showing various aspects of TB data reported to the CDC is available through NTSS to authorized state and local TB program staff. Information provided through NTSS reports includes the numbers of missing and unknown values associated with reported data elements, the frequency of reporting for select elements, when data were last transmitted to the CDC, and a list of elements with no information ever reported for a particular reporting area. State and local TB program staff can use these reports to identify and correct gaps in reported data or to report data errors to the CDC. The National Tuberculosis Indicators Project (NTIP) can also be used to verify and check TB surveillance data reported to the CDC [13]. Reporting jurisdictions can compare their records with NTIP data and use the NTIP to identify discrepancies. The RVCT has an accompanying manual that provides comprehensive reporting guidance for each data element [15]. Furthermore, the RVCT workgroup, composed of CDC and state and local TB program staff, actively pursues clarification and provides guidance on improving RVCT reporting. As state and local TB control programs are often challenged with declining resources and staff turnover, the CDC should periodically provide updated quality assurance and RVCT training webinars and materials to ensure that TB control program staff remain aware of data problem areas and new and existing quality assurance tools and techniques. These efforts, as well as ongoing discussions regarding data quality assurance, will improve the completeness and accuracy of TB surveillance data.

State and local communicable disease surveillance systems vary from disease-specific systems to systems used for reporting an array of diseases and conditions [9]. However, from 2007 to 2010, interoperability and integration of state and local public health disease surveillance systems increased substantially [9]. As public health programs begin to utilize current advances in electronic reporting and embrace new national guidelines related to health information exchange and meaningful use, more electronic surveillance systems will be modified to increase capacity and meet national standards [9,16]. The results of the NTSS transition from a single, stand-alone surveillance system to a variety of different reporting schemes illustrate that major modifications of disease surveillance systems can be done without substantial impact on the completeness of surveillance data.

## Abbreviations

CDC: Centers for Disease Control and Prevention
eRVCT: electronic Report of Verified Case of Tuberculosis
NEDSS: National Electronic Diseases Surveillance System
NTIP: National Tuberculosis Indicators Project
NTSS: National Tuberculosis Surveillance System
RVCT: Report of Verified Case of Tuberculosis
TB: tuberculosis
TIMS: Tuberculosis Information Management System

## References

[1] World Health Organization (2013). Global Tuberculosis Report.

[2] Sprinson JE, Lawton ES, Porco TC, Flood JM, Westenhouse JL (2006). Assessing the validity of tuberculosis surveillance data in California. BMC Public Health.

[3] Public Health Service (US) (1953). Reported tuberculosis morbidity and other data. Reported Tuberculosis Data 1953-69.

[4] Centers for Disease Control and Prevention (US) (1986). 1985 Tuberculosis statistics, states and cities. Tuberculosis Statistics in the United States, 1985, 1985-1986, 1987, 1988.

[5] Centers for Disease Control and Prevention (US) (1993). Reported Tuberculosis in the United States.

[6] Centers for Disease Control and Prevention (US) (2012). Reported Tuberculosis in the United States, 2012.

[7] Centers for Disease Control and Prevention (US) Public Health Information Network.

[8] Centers for Disease Control and Prevention (US) National Notifiable Diseases Surveillance System (NNDSS).

[9] Centers for Disease Control and Prevention (US) (2011). State electronic disease surveillance systems—United States, 2007 and 2010. MMWR Morb Mortal Wkly Rep.

[10] National Public Health Service and Biosurveillance Registry for Human Health (US) (2011). Tuberculosis Information Management System (TIMS).

[11] Winston CA, Navin TR, Becerra JE, Chen MP, Armstrong LR, Jeffries C, Yelk Woodruff RS, Wing J, Starks AM, Hales CM, Kammerer JS, Mac Kenzie WR, Mitruka Kiren, Miner Mark C, Price Sandy, Scavotto Joseph, Cronin Ann M, Griffin Phillip, LoBue Philip A, Castro Kenneth G (2011). Unexpected decline in tuberculosis cases coincident with economic recession—United States, 2009. BMC Public Health.

[12] Magee E, Tryon C, Forbes A, Heath B, Manangan L (2011). The National Tuberculosis Surveillance System training program to ensure accuracy of tuberculosis data. J Public Health Manag Pract.

[13] Manangan LP, Tryon C, Magee E, Miramontes R (2012). Innovative quality-assurance strategies for tuberculosis surveillance in the United States. Tuberc Res Treat.

[14] Centers for Disease Control and Prevention (US) (2009). Quality assurance for tuberculosis surveillance data: A guide and toolkit.

[15] Centers for Disease Control and Prevention (US) CDC tuberculosis surveillance data training: Report of Verified Case of Tuberculosis (RVCT) instruction manual.

[16] Shapiro JS, Mostashari F, Hripcsak G, Soulakis N, Kuperman G (2011). Using health information exchange to improve public health. Am J Public Health.

